# Whole-Body SPECT/CT: Protocol Variation and Technical Consideration—A Narrative Review

**DOI:** 10.3390/diagnostics14161827

**Published:** 2024-08-21

**Authors:** Mansour M. Alqahtani

**Affiliations:** 1 Department of Radiology and Medical Imaging, Faculty of Applied Medical Sciences, Najran University, Najran 61441, Saudi Arabia; mmalqahtane@nu.edu.sa; 2 Discipline of Medical Imaging Science, Faculty of Medicine and Health, The University of Sydney, Sydney, NSW 2006, Australia

**Keywords:** SPECT/CT, whole-body SPECT/CT, planar bone scintigraphy, bone metastases, acquisition protocol

## Abstract

Introducing a hybrid imaging approach, such as single-photon emission computerized tomography with X-ray computed tomography (SPECT)/CT, improves diagnostic accuracy and patient management. The ongoing advancement of SPECT hardware and software has resulted in the clinical application of novel approaches. For example, whole-body SPECT/CT (WB-SPECT/CT) studies cover multiple consecutive bed positions, similar to positron emission tomography-computed tomography (PET/CT). WB-SPECT/CT proves to be a helpful tool for evaluating bone metastases (BM), reducing equivocal findings, and enhancing user confidence, displaying effective performance in contrast to planar bone scintigraphy (PBS). Consequently, it is increasingly utilized and might substitute PBS, which leads to new questions and issues concerning the acquisition protocol, patient imaging time, and workflow process. Therefore, this review highlights various aspects of WB-SPECT/CT acquisition protocols that need to be considered to help understand WB-SPECT/CT workflow processes and optimize imaging protocols.

## 1. Introduction

The last two decades have witnessed the emergence and rapid clinical adoption of hybrid positron emission tomography with computed tomography (PET/CT) over PET-only devices. The primary driver of this adoption is the ability of PET/CT to generate functional and morphologic correlative images that can enhance diagnostic accuracy [1]. Similar advancements are also being observed for single-photon emission tomography with CT (SPECT/CT), an approach that is fast transitioning from a rather underutilized technological option to a recognized position for enhancing the diagnostic competencies of single-photon imaging, with possible implications for the management of patients [2]. The usage of hybrid imaging techniques, including PET/CT, PETs with magnetic resonance imaging (PET/MR) and SPECT/CT are rapidly growing. They provide three-dimensional (3D) functional and anatomical information, which improves sensitivity and specificity compared to planar bone scintigraphy (PBS) [3]. In addition, the use of transmission-derived anatomical data enables the modeling and correction of certain radiation transport events, such as the attenuation of photons, to be incorporated into the reconstruction algorithm for improvements in both qualitative and quantitative data. Advances in imaging technologies are aimed at improving diagnostic accuracy and confidence in clinical practice. For example, total-body (TB) PET, with its large axial field of view, enables the imaging of the entire human body simultaneously. Also, using time of flight (TOF) PET improves lesion detection and image quantification, and reduces scanning time [4]. Furthermore, SPECT quantification is becoming practicable in multi-center and multi-vendor [5,6].

Recently, the novel approach of whole-body (WB) SPECT/CT has emerged, encompassing numerous successive bed positions equivalent to the acquisition of PET/CT [2]. Clinical evidence suggests that WB-SPECT/CT is more effective than planar bone scintigraphy (PBS) to evaluate bone metastasis (BM) and investigate suspicious lesions [7,8,9]. Consequently, WB-SPECT/CT may substitute PBS scintigraphy. Nevertheless, its lengthy scan time and the non-uniform protocols used in bone procedures are considered new challenges and concerns [2,10]. At the same time, the majority of commercial vendors offer improved reconstruction software that speeds up SPECT acquisition while keeping similar image quality, allowing for multi-field of view (FOV) SPECT/CT acquisitions [7,11]. Therefore, this article aims to investigate the acquisition protocols and types of reconstruction utilized for WB-SPECT/CT imaging, which can then inform the possibility of optimizing acquisition protocols to reduce scanning time while maintaining image quality.

### Advantages and Pitfalls

SPECT/CT has several advantages over PBS images, the most significant of which are enhanced attenuation correction, increased specificity, localized metabolic abnormalities to anatomical structures, and potential impacts on adjacent tissues. Moreover, when using SPECT/CT, it has been shown that the location and characteristics of endocrine and neuroendocrine tumors can be accurately determined, as well as solitary pulmonary nodules, lung cancers, lymphomas, brain tumors, and prostate cancers, as well as benign bone lesions, malignant bone lesions, and localized infection [12,13].

The widespread adoption and application of SPECT/CT in clinical practice is a further advantage of its use. However, the time required to obtain the entire axial skeleton to be imaged is a significant limitation that must be considered. This can cause significant patient discomfort and reduce departmental output. Long scan times can increase patient movement, which is especially problematic when acquiring multiple bed positions [10]. In addition, the required scan range will cause the CT radiation dosage to rise.

However, several studies show that using low-dose CT methods can reduce radiation exposure to an acceptable level, particularly when incorporating vendor software for automated exposure control. A study by Löfgren et al. [14] stated that the approximate radiation exposure is 4–5 mSv from a low-dose CT incorporated in the WB-SPECT/CT examination from the head to the mid-thigh. Other studies reported that the estimated radiation exposure for CT from the skull to the proximal femurs is 4.2 ± 1.0 mSv using ImPACT CT Patients Dosimetry Calculator (version 1.0.4, Imaging Performance Assessment on Computed tomography, www.impactscan.org, accessed on 21 June 2024)) [15,16]. Meanwhile, Fleury et al. [8] suggest that irradiation caused by morphologically modulated, low-dose CT performed for WB-SPECT/CT can be considered acceptable with an effective dose of around 6 mSv.

## 2. WB-SPECT/CT Acquisition Protocols and Technical Considerations

Acquisition protocols and reconstruction parameters of the WB-SPECT/CT studies published in the literature are summarized and listed in Table 1, including the scanner type, radiotracer, administration dose, and body range scanned. The SPECT/CT scanners used in the studies varied in model, including thirteen Siemens (Symbia T2, T6, T16, E-CAM and Intevo 6), nine GE Healthcare (Hawkeye 4 Infinia or Varicam and Discovery NM/CT 670) and two Precedence models (Philips Healthcare). There were nine studies that reported using hydroxy diphosphonate/methylene (HDP/HMDP) and seven using MDP, with one exceptional study that used diphosphono-1,2-propanodicarboxylic acid (DPD) [17]. The administration dose range was 523–1000 MBq with a mean ± standard deviation value of 735.5 ± 120. A recent study stated that the radiation effective dose caused by the 99mTc-HMDP scintigraphy is 3.8 mSv [18]. This number is close to the diagnostic reference levels DRLs (3.82 mSv) considering the mean injected activity is below the ICRP limit (750 MBq) [19]. Nonetheless, the effective dose of this dose is largely determined by the patient’s weight and the type of radiopharmaceutical administered.

**Figure 1:**
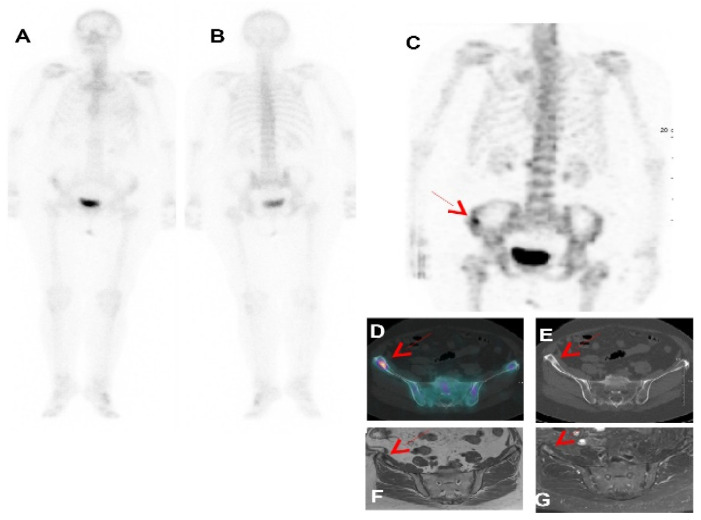
Patient with PBS Sc 3 and WB-SPECT/CT Sc 1 proved as a true positive by follow-up. In a 79-year-old female with newly diagnosed breast cancer with involved lymph node (T1N1), PBS in ventral (**A**) and dorsal (**B**) projections did not reveal any suspicious bone lesion. Right iliac aisle uptake (red arrow) was seen on (**C**) trunk SPECT/CT MIP (maximum intensity projection) and (**D**) fusion SPECT/CT with a sclerosis lesion observed on CT (**E**). Bone metastasis was confirmed using MRI and fat-saturated T1 and T1 gadolinium (**F**,**G**) and clinical follow-up [8].

**Figure 2:**
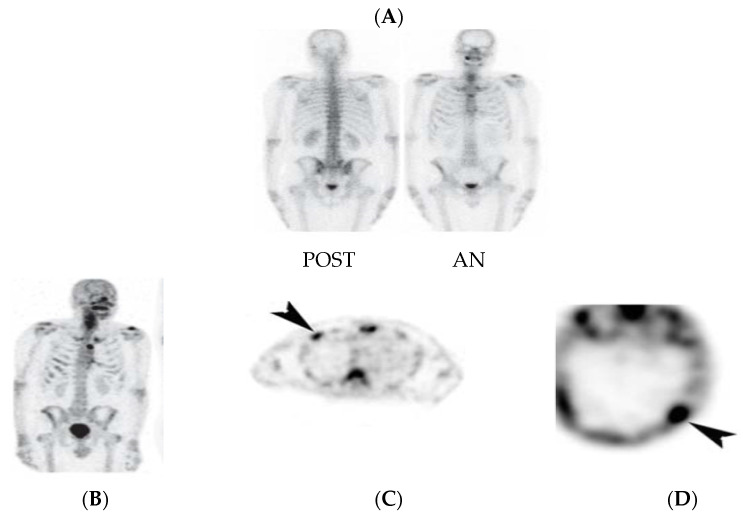
Early metastatic dissemination that has been on planar BS for a 57-year-old patient with prostate cancer. Image (**A**) shows the posterior and anterior planar BS, (**B**) shows the multi-FOV SPECT. (**C**) PBS was found to be negative on SPECT (osteoblastic rib metastasis). (**D**) Osteoblastic metastasis in skull on SPECT (metastasis marked by arrowhead) [7].

SPECT/CT imaging protocols included two or more axial FOV tomographic acquisitions that covered the skull vertex to the mid-thigh or the cervical region to the proximal femur. The gamma cameras employed in many studies had an axial FOV of 38.7 cm, which corresponds to a total axial FOV of 112 cm for three SPECT acquisitions, including a 2 cm overlap for sequential bed positions [14,15,16]. Many studies suggested that three bed positions from the vertex to the mid femur with around 112 cm would be sufficient unless otherwise clinically required in specific cases. Indeed, WB-SPECT/CT significantly increased the number of observed metastases that could have been missed on a PBS [7,8,20,21,24]. Moreover, as shown in Figure 1, the study found that lesion detection using SPECT/CT was improved for lesions situated in the lower thoracic and lumbar vertebral column [31,32,33]. Additionally, it is argued by Even-Sapir et al. [7] that the performance of WB-SPECT/CT on the whole axial skeleton enables metastases to be detected in other areas, including the skull, rib cage, upper vertebral column, long bones, and pelvis (see Figure 2).

**Figure 3:**
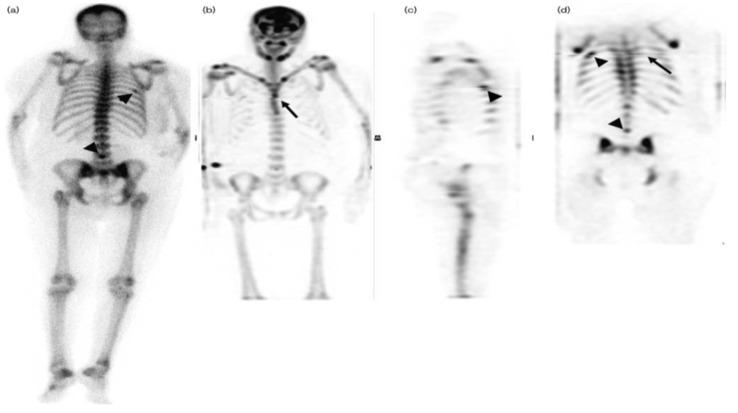
Planar BS image showing benign rib has been missed on WB-SPECT due to the presence of a reconstruction artefact (**a**). Posterior PBS highlights focal tracer uptake in the right 6th rib and spinous process of L4 fractures (arrowheads). This image is of a breast cancer and renal osteodystrophy patient. The rib finding was classified as an artefact on WB-SPECT ((**b**) MIP, (**c**) selected sagittal, and (**d**) coronal slices, arrowheads) due to SPECT reconstruction artefact at the overlap of the first and second SPECT FOV (arrows) caused by slight patient movement with subsequent ill-defined activity at the level of the zip artefact that could not be prospectively characterized as a true lesion. BS, bone scintigraphy; FOV, field of view; MIP, maximum intensity projection; WB-SPECT, whole-body single-photon emission computed tomography [22].

When interpreting WB-SPECT/CT, reporting physicians must be mindful of the possibility of artefacts. Multi-FOV SPECT can sometimes overlap to smooth the transition, and this overlap is automatically pasted together by image reconstruction software. Sometimes a slight zip line can be seen at the fusion site. Usually, this does not affect interpretation so long as the patient remains still. However, if the patient moves, this can be inadvertently overlooked (note the overlooked rib fracture in the WB-SPECT study presented in Figure 3 [22]. It is also better if the injection site is outside the FOV, as this can eliminate the reconstruction artefacts caused by interstitial injections.

The acquisition times for WB-SPECT/CT protocol for the GE healthcare system, Siemens Medical Solution, and the Philips Healthcare system ranged from 22 to 75 min for total examination time incorporating the use of low-energy high-resolution (LEHR) collimators. However, the number of projection angles were varied, as demonstrated in Table 1. The majority of the articles used ordered subset expectation maximization (OSEM) with distance-dependent resolution modeling and RR for the image reconstructions (Table 2).

The OSEM algorithm is a modified version of the maximum likelihood expectation maximization (MLEM) strategy that partitions the collected projection data into subsets to minimize computational load [34]. Even though different effects can be modeled using MLEM (i.e., scatter elimination, system response, and photon attenuation correction), the primary issue with the MLEM algorithm is that as the number of iterations increases, image noise increases, which is even more prominent in low-count acquisitions [35]. Meanwhile, in resolution recovery (RR) methods, the three-dimensional collimator response in image production is taken into account, which reduces the impact of the point-spread function (PSF) on image resolution. This method can thus be extremely useful when noise reduction is essential for ensuring image quality in low-count studies. However, such correction shaves been known to introduce Gibbs, or ringing, artefacts in the reconstructed data around areas of high contrast. Notably, the RR OSEM reconstruction approach applies collimator detector response (CDR) to reconstructed SPECT images by incorporating intrinsic response, geometric penetration, septal scatter, and penetration components [36].

Image noise in CT, which degrades CT image quality, is inversely related to the X-ray beam energy and increases as tube current or tube voltage decreases. The CT X-ray tube current-time product, as reported in the previously quoted WB-SPECT/CT articles, ranged from 2.5 to 100 mAs. Nonetheless, the literature evidence suggests that low-tube current-time products ranging between 2.5 to 30 mAs can be used to decrease radiation dose while maintaining adequate CT image quality for attenuation correction purposes and basic anatomical localization [37]. Palmedo et al. [21] reported that using a low current-time product of 2.5 mAs resulted in an effective dose of 0.4 mSv and caused a significant improvement in diagnostic precision and patient management without significantly increasing the overall radiation dosage to the patient. In addition, to minimize radiation exposure, Rager et al. [15] recommend reducing the tube current to 40 mAs and activating the intensity modulation tool (CARE Dose 4D, Siemens Healthcare).

## 3. Evidence on the Role of WB-SPECT/CT in Imaging of Bone Metastases

There is published literature evaluating WB-SPECT/CT’s role in detecting BM on a per-patient or per-lesion basis compared to PBS. WB-SPECT/CT had a superior accuracy in BM detection and more reliable performance with less equivocal results (2.8% vs. 20%). (See the review for more details) [9].

Abikhzer et al. [22], in a retrospective study, demonstrated the clinical utility of WB-SPECT compared to PBS for the BM detectability in breast cancer patients. In this study, WB-SPECT lesion detection (100%) was not statistically different from PBS (97%) performance in a patient-based analysis. However, statistically significant differences were seen in a lesion-based analysis with PBS detection (80%), falling well below WB-SPECT (100%) outcomes (*p* < 0.001). Furthermore, confidence levels were higher for WB-SPECT in comparison with PBS for both benign (*p* < 0.01) and malignant (*p* < 0.05) lesions.

Higher diagnostic confidence is gained with WB-SPECT/CT when compared to PBS. The diagnostic confidence of WB-SPECT/CT in breast and prostate cancer patients was supported by the reported significant reduction in suspicious findings characterization (2.5%) compared to PBS (28.7%) [21]. Moreover, the anatomical site of an equivocal lesion was not truly indicated on PBS and/or SPECT images but was on WB-SPECT/CT images; through WB-SPECT/CT, new tumor sites were identified and false-positive lesions were identified as true-negatives on PBS and/or SPECT images. The most commonly identified lesions were located in the spine (448) and pelvis (211). However, a significant number of lesions were found in other skeletal areas including the ribs, shoulders, sternum, femora, and cranium (180) [21]. Furthermore, among 80 patients with various primary cancers, 151 indeterminate lesions on PBS were characterized in WB-SPECT/CT. The improvement was mostly (135 lesions) located in the axial skeletal [26]. Therefore, these outcomes demonstrate the usefulness of WB-SPECT/CT imaging in the detection of axial skeletal lesions.

In another work by Guezennec et al. [17], the authors reached an agreement with a previous published study by Palmedo et al. [21], concluding that the testing of the acquisition protocol of WB-SPECT/CT for the initial staging of patients with cancer had limited incremental diagnostic value compared to the commonly used PBS and targeted SPECT/CT strategy in regard to specificity. However, the tested protocol showed slightly enhanced sensitivity in unseen lesions on PBS, which could be advantageous in initial staging activities, thereby supporting the clinical utility of the addition of SPECT/CT to PBS.

Furthermore, research involving 194 patients with prostate carcinoma showed that, in patient-based analysis, the diagnostic accuracy for the WB-SPECT/CT examinations were higher than for PBS, particularly in patients who had serum PSA levels under 40 ng/mL. Nonetheless, there was no difference in the sensitivity of PBS and WB-SPECT/CT in patients with PSA levels higher than 40 ng/mL. Thus, carrying out multi-FOV SPECT/CT acquisitions on prostate carcinoma patients with PSA levels under 40 ng/mL could allow a reduction in the gamma camera workload whilst maintaining the diagnostic advantages of multi-FOV SPECT/CT in challenging patients [20]. Additionally, SPECT/CT will be useful in cases where CT is insufficient, especially in distinguishing between BM and early avascular necrosis [38].

## 4. WB-SPECT/CT vs. PET/CT

PET/CT has been used with different tracers for the detection of breast and prostate cancer metastases, such as Fluorine-18 fluorodeoxyglucose (18F-FDG) and sodium fluoride (18F-NaF). In particular, 18F-NaF PET/CT is superior to the SPECT/CT [24] but, with regard to 18F-FDG PET/CT, a study by Rager et al. [16] on breast cancer patients demonstrated, in a lesion-based analysis, a higher diagnostic accuracy of WB-SPECT/CT (*p* = 0.004) during follow-up (n = 21 months). In contrast, WB-SPECT/CT should be used in conjunction with 18F-FDG PET/CT to identify non-bone extra-axillary nodal involvement and distant metastases.

Another study by Abikhzer et al. [23] provided a prospective comparison of WB-SPECT using 99mTc-MDP for BM detection in breast cancer patients in comparison with 18F-NaF PET performance. During patient-based analysis, both methods succeeded in detecting 19 of the 21 metastases with the 18F-NaF PET modality distinguished by its detection of a single metastasis found in two patients. Overall WB-SPECT performance demonstrated adequate sensitivity (63%), specificity (97%), PPV (89%), and NPV (87%). During lesion-based analysis, WB-SPECT detection (60%) was well-below 18F-NaF PET performance (94%); overall, 18F-NaF PET sensitivity (95%) was significantly higher (32%) than WB-SPECT sensitivity (65%) (*p* < 0.001), specificity (97%), PPV (89%), and NPV (87%).

In the study by Dyrberg et al. [39], a prospective study of BM detection in prostate cancer patients, all three nuclear medicine methods (WB-SPECT/CT, NaF-PET/CT, and choline-PET/CT) and WB-MRI performed similarly in all patient-based diagnostic measures with WB-SPECT/CT sensitivity (100%), specificity (91%), and accuracy (94%) reporting higher than WB-MRI at 89%, 90%, and 90%, respectively. Like Jambor et al. [24], Dyrberg et al.’s [39] protocol used 99mTc-HDP in a similar coverage area but at a slightly higher average dose (717 MBq) and in a different cancer type target. In the study by Dyrberg et al. [39], total acquisition time (75 min) was more than double that of Jambor et al. [24] (30 min). Also, WB-SPECT/CT specificity (88%) fell by 6% in patients receiving androgen-deprivation therapy (ADT), which was 12% lower than SPECT/CT specificity performance in Fonager et al.’s study [25],which may be explained by study sample differences, although both sensitivity and specificity were statistically similar in both studies.

A recent study by Bénard et al. [40] demonstrated the superiority of 18F-NaF PET-CT over 99mTc-MDP SPECT in detecting bone metastases (BM). This prospective, multicenter, phase 3 trial included 261 patients with high-risk prostate or breast cancer. The findings revealed that 18F-NaF PET-CT was significantly more accurate than 99mTc-MDP SPECT (84.3% vs. 77.4%, *p* = 0.016) in identifying bone metastases. Additionally, 18F-NaF PET-CT exhibited higher sensitivity (78.9% vs. 63.3%, *p* = 0.0007), negative predictive value (85.4% vs. 76.9%, *p* = 0.0006), and positive predictive value (82.7% vs. 78.4%, *p* = 0.38), while maintaining similar specificity (88.2% vs. 87.5%, *p* = 0.86). Therefore, these findings were consistent across both prostate and breast cancer subgroups, suggesting that 18F-NaF PET-CT could potentially replace 99mTc-MDP as the bone imaging radiopharmaceutical of choice in these high-risk populations.

Whilst PET has superior accuracy and spatial resolution over SPECT, which may play a role in the detection of small lesions, cost-effectiveness and availability are major considerations in modality choice when comparing both modalities. The effective dose to the patient from 18F-NaF PET/CT varies with the administered activity. Current guidelines suggest a dose range of 185–370 MBq [41]. An effective dose of 10 mSv is associated with 370 MBq of 18F-NaF, which is higher than the 5.3 mSv dose from 925 MBq of 99mTc-MDP [42]. However, the CT dose in PET/CT and SPECT/CT systems is approximately equivalent over the same axial range [14]. Thus, the difference in radiation dose is attributed solely to the radiopharmaceuticals used. However, given the high bone-to-background ratio (i.e., high signal-to-noise ratio) of 18F-NaF, it is possible to reduce the injected activity (e.g., by approximately 50%) without significantly compromising image quality. This reduction would lower the effective dose to a level comparable to that of 99mTc-MDP [43].

## 5. Developments in WB-SPECT/CT Protocol

Iterative reconstruction methods using correction for attenuation, scatter, and CDR can shorten bone SPECT acquisition times without negatively affecting image quality or diagnostic accuracy. Study findings support the use of a WB-SPECT-based protocol to improve existing bone scan best practices, including the possible justified elimination of initial PBS [22]. Another study by Even-Sapir et al. [7] also reported similar advantages of using CDR. They reported that the total acquisition time of multi-FOV SPECT imaging is within 24–32 min using CDR, by which a quantitative model of the CDR function of the acquisition system is integrated into the reconstruction algorithm in order to minimize collimator detection blur effects and therefore enhance image resolution and signal-to-noise characteristics. Furthermore, iterative reconstruction techniques integrating RR have recently been introduced, which have the potential to minimize imaging times while maintaining image quality. Reconstruction with RR attempts to correct characteristics of the imaging system that act to degrade the final image (primarily collimator geometry and detector response), thereby producing reconstructed images that show improved image quality compared with conventional SPECT [44,45].

Due to recent advances in technology, new SPECT/CT machines have been developed that can sequentially cover and accurately merge multiple bed positions. It was noted that studies used gamma camera systems, and specially-designed WB-SPECT software covering all of the SPECT fields was employed to scan the whole SPECT/CT area from the cervical region to the proximal femora [20,21]. In addition, many new methods are being continuously developed for SPECT but have not yet been integrated into standard clinical practice. Examples include cadmium–zinc–telluride (CZT) detectors, list-mode SPECT acquisitions, multi-pinhole collimators, novel non-parallel hole collimators, and radiopharmaceutical quantification. It is anticipated that deep learning techniques will be utilized in nuclear medicine imaging in the future [46]. Efforts should be increased to standardize acquisition protocols.

While multiple protocols exist for SPECT/CT, including both targeted SPECT/CT and WB-SPECT/CT, the literature synthesis indicates the importance of WB-SPECT/CT to augment PBS, as it can reduce equivocal findings and improve user confidence [2,21,26,28]. In addition, since many routine bone procedures involve planar plus one or two bed SPECT/CT over the area of interest, the application of fast WB-SPECT/CT is likely to enhance patient comfort, increase throughput, and decrease motion artefact [2]. Therefore, the following question must be asked: can fast WB-SPECT/CT replace PBS in cancer patients? Recently, manufacturers such as GE and Spectrum Dynamics introduced a new SPECT-only system design that can enable WB imaging in a short acquisition time with enhanced tomographic spatial resolution [47]. However, the current conventional gamma camera system will continue to be the “workhorse” for some years, and we need WB-SPECT even though not all departments will have one of these new scanners. Therefore, optimizing the acquisition protocol to reduce acquisition time while maintaining image quality is required.

Molecular imaging in oncology has generally shifted away from the utilization of SPECT/CT toward PET/CT due to the better resolution, sensitivity, quantification, and lesion contrast [42,48,49,50]. However, imaging with single-photon emitting nuclides continues to be widely used, providing affordable and clinically relevant information in oncologic and non-oncologic indications [51,52,53,54,55]. In addition, continuous advancements in gamma camera technology, reconstruction, and detector system modeling have also enabled quantitative SPECT imaging [56]. A recent article “Quantitative SPECT/CT: SPECT joins PET as a quantitative imaging modality” by Bailey et al. [57] highlights the potential for clinical applications of quantitative measurements utilizing SPECT/CT following the widespread adoption of SPECT/CT systems. The authors have demonstrated the advantages of quantitative SPECT over PET, including the ability to perform multi-tracer studies simultaneously using various radionuclides, and the longer physical half-lives of radionuclides which allow for the imaging of extended biological processes. Dickson et al. [5] revealed that quantitative SPECT provides meaningful metrics for longitudinal assessments of disease progression and post-radionuclide therapy dosimetry, but its inter-patient use cases require further evaluation to determine clinical value. Another review on quantitative SPECT, “Does quantification have a role to play in the future of bone SPECT”? authored by Ross et al. [58] stated that the availability of WB-SPECT/CT for quantitative assessments might provide additional insight into extent of disease before and after radionuclide therapy. As a result, SPECT-based quantitative imaging has gained increased significance in clinical practice.

Recent advancements in detector technology, particularly the introduction of CZT detectors, are enabling more widespread WB-SPECT imaging [59]. CZT technology offers several advantages over traditional sodium iodide (NaI) scintillation crystals. Firstly, CZT detectors provide superior energy resolution, typically in the range of 5–6%, compared to 9–10% for NaI crystals. This enhancement allows for better discrimination between primary photons and scattered radiation, resulting in improved image contrast and reduced noise. Secondly, CZT detectors offer higher sensitivity through the direct conversion of gamma rays to electrical signals, eliminating the need for photomultiplier tubes. This improvement leads to increased detection efficiency and sensitivity, potentially enabling the use of lower injected doses or shorter acquisition times. The pixelated nature of CZT detectors also contributes to improved intrinsic spatial resolution, further enhancing image quality [60,61].

SPECT systems utilizing CZT have demonstrated the ability to perform WB-SPECT/CT in under 20 min, as reported by Gregoire et al. [62] and Arvola et al. [63]. The enhanced count rate capabilities of CZT detectors make dynamic SPECT imaging more feasible, opening new possibilities for functional imaging studies [64]. Moreover, the superior energy resolution of CZT detectors allows for better separation of different isotopes, facilitating more accurate simultaneous multi-isotope imaging [65]. Finally, CZT technology, when combined with advanced reconstruction algorithms, improves the quantitative accuracy of SPECT imaging, bringing it closer to PET in terms of quantitative capabilities.

As CZT technology becomes more widely available, it is expected to have a significant impact on clinical protocols. The potential for faster acquisitions could improve patient comfort and increase throughput, while the ability to reduce injected doses aligns well with the as low as reasonably achievable (ALARA) principle in radiation protection. Future research should focus on optimizing acquisition protocols for CZT-based WB-SPECT/CT systems and evaluating their impact on diagnostic accuracy and patient outcomes in various clinical scenarios.

The generation of synthetic planar images from WB-SPECT data may increase the adoption of WB-SPECT and facilitate the transition from PBS. This approach enables the creation of 2D images without requiring additional time for planar acquisition and allows for multiple planar views through a full 360° rotation, offering a more accurate representation of SPECT images compared to the current maximum intensity projection (MIP) method [66]. Many studies have investigated the use of synthetic planar images from SPECT data, particularly in lung imaging. These investigations have focused on novel algorithmic methods [67] or new camera types, such as CZT detectors [59]. In a prospective study, synthetic images were generated from resampled SPECT data using list mode. Angular-summed and reprojected planar images were produced by summing projections over a 12° angular range for multiple perspectives and constructing a 3D synthesized attenuation map of the lungs and torso. This attenuation map was used to generate views corresponding to traditional planar imaging, with in-house software resampling and formatting of the images to match the pixel size of true planar scans [68]. The results demonstrated that R-PBS significantly enhances the reading of SPECT/CT, making this technique valuable in providing 2D images to aid clinicians, especially during the transition to 3D-only data due to their historical familiarity with 2D images.

Based on a thorough literature review and considering the balance between image quality and acquisition time, we recommend the following for an optimal WB-SPECT/CT protocol:

The optimization of reconstruction parameters requires a careful trade-off between noise levels and acquisition time. Our recent study suggests that rapid scanning, approaching 8 s per bed position with a low number of iterations (4–8) and 8 subsets, could be feasible and provides an optimal balance between acquisition time and image quality for WB bone SPECT/CT [69].

## 6. Future Work

WB-SPECT/CT can reduce equivocal findings, enhance user confidence, and potentially improve patient management. However, longer scan times negatively affects patient throughput and results in greater patient motion during the study due to reduced patient comfort and compliance. Nevertheless, there is a variation in recommendations and findings because there is a lack of standardized guidance for WB-SPECT/CT bone procedures. Hence, it is suggested that future research should focus on WB-SPECT/CT to assess acquisition and reconstructions by specialist review and comparison. This could be achieved by using a gated dataset along with an ECG trigger or list mode and proportion of the time-bin reconstruction for the clinical optimization of WB-SPECT/CT acquisition and achievement of equilibrium between image quality and time. Such an approach could permit the data and generation of acquisitions that have any percentage of counts that belong to the full-time dataset without increasing the number of necessary scans. Additionally, ensuring the reproducibility of these examinations across different SPECT/CT devices would be an interesting exercise to confirm that the proposed approach applies to any SPECT/CT scanner.

## 7. Conclusions

WB-SPECT/CT appears to be a promising imaging method for BM detection. Standardization of protocols and additional research are required to optimize the impact of such technology in clinical practice.

## Figures and Tables

**Table 1:** Summary of acquisition protocol of WB-SPECT/CT.

Author, Year	Scanner (Vendor)	Patients Number	Radiotracer	Admin. Dose, MBq	Body Part Covered	SPECT Parameters					CT Parameters				Results
						Total Number of Projections	Time /Projection (Sec/View)	Acquisition Duration (Min)	Post-Filtering	Reconstruction Methods	Scan Slice Thickness (mm)	Radiation Dose in mAs (kV)	Radiation Dose (mSv)	CT Slice Used	
Even-Sapir, 2006 [7]	Discovery VH (GE Healthcare)	24	^99m^Tc-MDP	925	Entire axial skeleton	/	/	24–32	/	Xeleris workstation (GE Healthcare)	/	80 Care4D (140)	/	/	Diagnosis: prostate cancer. In patient-based analysis: WB-SPECT/CT’s sensitivity 92%, specificity 82%. In lesions-based analysis: sensitivity and specificity improved from 39% and 79% for PBS to 71% and 85% for WB-SPECT. WB-SPECT values were significantly increased compared to PBS (*p* < 0.05). Sensitivity of 18F-Fluoride PET significantly higher than WB-SPECT (*p* < 0.05).
Giovanella, 2011 [20]	E-CAM (Siemens Medical Solution)	194	^99m^Tc-HDP	740	Vertex of the skull to mid-femur	64	18	28.8	Gaussian function with cut-off 8 mm.	3D OSEM (8/10)	/	/	/	/	Diagnosis: prostate cancer with PSA levels >10 ng/mL. In patient-based analysis: sensitivity 95%, specificity 96%. In lesions-based analysis: sensitivity 98%, specificity 93%. WB-SPECT had superior specificity than PBS (*p* < 0.01).
Palmedo, 2013 [21]	i. Hawkeye 4 Infinia, GE Healthcare ii. Symbia T2, (Siemens Medical Solution)	308	^99m^Tc-MDP	700	Cervical region to the proximal femur	64	25	25	/	Special high-resolution algorithm	2.5–5	2.5–20 (120)	0.4	2 and 4	Diagnosis: prostate and breast cancer. Sensitivity 97.2%, specificity 94.1%. Total of 36 patients converted from false-positive by PBS to true-negative using WB-SPECT/CT (downstaging rate 32.1% of total group).
Abikhzer, 2015 [22]	Discover NM/CT 670 or infinia Hawkeye 4 (GE Healthcare)	92	^99m^Tc-MDP	925	Skull vertex to the mid-thigh	60	17	28 (including detector positioning)	Smoothed with a 3D spatial Gaussian filter (2.0 mm at FWHM)	3D OSEM (6/10)	/	/	/	/	Diagnosis: breast cancer. Out of 268 lesions, WB-SPECT detected 266, whereas PBS detected 195 lesions. WB-SPECT values of confidence were significantly higher compared to PBS for both benign (*p* < 0.01) and malignant lesions (*p* < 0.05).
Abikhzer, 2016 [23]	Discover NM/CT 670 or infinia Hawkeye 4 (GE Healthcare)	41	^99m^Tc-MDP	925	Skull vertex to the mid-thigh	60	17	28 (including detector positioning)	Smoothed with a 3D spatial Gaussian filter (2.0 mm at FWHM)	3D OSEM (6/10)	/	/	/	/	Diagnosis: breast cancer. When compared to WB-SPECT imaging, 18F-PET had the highest sensitivity for the detection of BM in breast cancer, increasing by 10% on a patient-based analysis and 32% on a lesion-based analysis.
Jambor, 2016 [24]	Symbia T6, (Siemens Medical Solution)	53	^99m^Tc-HDP	670	Tip of the head to the mid-thigh	90	9	30	/	(5/10), (Siemens)	/	10 (130)	/	/	Diagnosis: prostate and breast cancer. WB-SPECT/CT having lesser equivocal finding compared to WB-SPECT and PBS. WB-MRI + DWI has comparable sensitivity to 18F-NaF PET/CT and superiority to WB-SPECT/CT in detecting BM.
Guezennec, 2017 [17]	i. Symbia Intevo 6 (Siemens Medical Solution) ii. Symbia T6 (Siemens Medical Solution)	102	^99m^Tc-DPD	9MBq/kg	Upper cervical spine to the proximal femora	120	10	20	Smoothed with a 3D spatial Gaussian filter (10.0 mm at FWHM)	3D OSEM (8/16)	/	90 Care4D (130)	5	6	Diagnosis: patients proven malignancy. In a per-lesion analysis: 91 and 241 equivocal and suspicious lesions, respectively, when PBS was used, 17 and 259 when single FOV SPECT/CT was used, and 11 and 269 when two FOV SPECT/CT was used. On a per-patient basis, the diagnosis was negative, equivocal, or suspicious for malignancy in 35, 53, and 14 patients using PBS, respectively, 77, 6, and 19 patients using an additional single FOV SPECT/CT image, respectively, and 76, 7 and 19 patients, respectively, using Tow FOV SPECT/CT images.
Rager, 2017 [15,17]	Symbia T6, (Siemens Medical Solution)	212	^99m^Tc-HMDP	732 ± 172	Skull to the proximal femurs	64	15	22	Smoothed with a 3D spatial Gaussian filter (1 mm at FWHM)	3D OSEM (8/8),	3	40 Care4D (110)		6	Diagnosis: various cancers. WB-SPECT/CT is more sensitive for detecting extra-axial lesions (*p* = 0.0266), notably in the femoral neck.
Löfgren 2017 [14]	i. Symbia T16 [Siemens Medical Solutions] ii.Precedence [Philips])	117	^99m^Tc-HDP ^99m^Tc-HDP	Range 523–655 Range 523–655	Head to the mid-thigh Head to the mid-thigh	180 128	6 8	30–35 30–35	/ /	(5/10) (4/16) Astonish	5 5	Weight adapted dynamic (130) Weight adapted dynamic (140)	/ 4–5	16	Diagnosis: various cancers. In comparison to PBS, 8F-NaF PET/CT and WB-SPECT/CT significantly reduced the number of equivocal outcomes. No comparison between two acquisition times used for WB-SPECT/CT.
Fonager, 2017 [25]	i. Symbia T16, (Siemens Medical Solutions) ii. Symbia T2 and T16 (Siemens Medical Solutions)	37	^99m^Tc-MDP ^99m^Tc-MDP	750–1000 10 MBq/kg	Vertex to mid-thigh Vertex to mid-thigh	64 128	20 10	32 32	/ /	Iterative algorithm Iterative algorithm	3 5	100 Care4D (130) 20 (130)	/ /	2 and 16	Diagnosis: prostate cancer with PSA levels ≥ 50 ng/mL (high-risk). Sensitivities and specificities for PBS were 78 and 90%, respectively; for WB-SPECT/CT, they were 89 and 100%, respectively. No comparison between acquisition times used for WB-SPECT/CT.
Teyateeti, 2017 [26]	i. Discover NM/CT 670 or infinia Hawkeye 4 (GE Healthcare) ii. Symbia T and Symbia E (Siemens Medical Solution)	80	^99m^Tc-MDP	740	Vertex to mid-thigh	60	15	22.5 (25 min with CT)	Post-reconstruction Butterworth filtering (0.48/10)	3D OSEM with high-frequency noise IR applied on a computer workstation (Xeleris, GE healthcare).	3.75	20 (120)	/	4 and 16	Diagnosis: various cancers. Diagnostic accuracy for PBS and WB-SPECT/CT were 60% and 99.8%, respectively. WB-SPECT/CT was performed complementary to the PBS.
Mahaletchumy, 2017 [27]	Infinia Hawkeye (GE Healthcare)	85	^99m^Tc-MDP	740	Skull to mid-thigh	60	15	22.5	Images were smoothed with Hann and Butterworth filter	3D OSEM (2/10)	5	2.5 (140)	/	1 and 4	Diagnosis: breast cancer. WB-SPECT/CT reduced the proportion of equivocal diagnoses on both a patient- and lesion- basis (*p* <0.004).
Fleury, 2018 [8]	i. Discovery NMCT670 (GE Healthcare) ii. Symbia T2 (Siemens Medical Solution)	328	^99m^Tc-HMDP	9MBq/kg	Skull to mid-thigh	90	10	25 for (fast BS + WB-SPECT/CT)	/	3D OSEM (4/8)	2.5 5	Modulation mA, (140) Modulation mA, (130)	6 6	2 and 16	Diagnosis: prostate and breast cancer. Total of 67 (20.4%) patients had equivocal lesions using PBS, whereas 6 (1.8%) patients had equivocal lesions using WB-SPECT/CT.
Mavriopoulou, 2018 [28]	i. Howkeye-Varicam (GE Healthcare) ii. Hawekey-4, Infinia (GE Healthcare)	257	^99m^Tc-HDP ^99m^Tc-HDP	630–700 630–700	Whole vertebral column to the proximal femur Whole vertebral to the proximal femur	60 60	15 12	25–40 25–40	Butterworth (0.45/10) Butterworth (0.45/10)	3D OSEM (2/10), and viewed on a “Xeleris, version 2 or 3” workstation	10 4.4	2.5 (140) 2.5 (140)	/ /	1 and 4	Diagnosis: breast cancer. WB-SPECT/CT’s sensitivity was 96.9%, specificity was 87.5%. After WB-SPET/CT, the interpretation of PBS changed in 74 (28.8%) patients. Axial FOV = 40 cm.
Rager, 2018 [16]	Symbia T6 (Siemens Medical Solution)	25	^99m^Tc-HDP	10 MBq/Kg	Skull base to the proximal femurs	64	15	24	Smoothed with a 3D spatial Gaussian filter (10.0 mm at FWHM)	3D OSEM (8/8) (Siemens)	3	40 Care4D, (110)	4.2 ± 1.0 mSv	6	Diagnosis: breast cancer. WB-SPECT/CT had a higher diagnostic accuracy (88%) than FDG PET/CT (56%) for the detection of BM on a per-lesion basis. WB-SPECT/CT plus FDG PET/CT was found to be superior to each modality alone for staging.
Dyrberg, 2018 [29]	Precedence (Phiips Healthcare)	109	^99m^Tc-HDP	717	Top of the skull to mid-thigh	/	/	75	/	Astonish	1.5	50 (120)	/	16	Diagnosis: prostate cancer. Aim of this study was to compare whole-body SPECT/CT, choline-PET/CT NaF-PET/CT, and WB-MRI including DWI. All three nuclear medicine modalities and WB-MRI performed well in detecting BM, with no statistically significant difference.
Arvola, 2019 [30]	Symbia T6 (Siemens Healthcare)	53	^99m^Tc-HDP	672 ± 21	Top of the skull to mid-thigh	180	9	40.5	Smoothed with a 3D spatial Gaussian filter (7.0 mm at FWHM)	3D OSEM (10/15) (HERMES Medical Solutions)	4.8	10 (130)		/	Diagnosis: prostate and breast cancer. The purpose was to obtain quantitative measures (SUVs) for both WB-SPECT/CT and 18F-NaF PET/CT. The strong correlation between SUVs of ^99m^Tc-HDP WB-SPECT/CT and 18F-NaF PET/CT indicates that WB-SPECT is an effective tool for clinical quantification of patients with breast or prostate cancer.

PSA, prostate specific antigen; SUV; standard uptake value; matrix size applied for all studies is 128 × 128.

**Table 2:** A summary of the recommended optimal acquisition protocol for WB-SPECT/CT.

Parameter	Recommendation
SPECT acquisition	
Energy window	15–20% centered at 140 keV
Matrix	128 × 128
Projections	60–120
Time per projection	8–20 s
Total SPECT time	20–30 min
CT acquisition	
CT type	Low dose
Tube current	30–50 mAs (modulated)
Tube voltage	120–130 kV
Slice thickness	2.5–5 mm
Reconstruction	
Algorithm	Iterative (e.g., OSEM)
Iterations/Subsets	2–4 iterations, 8–10 subsets
Corrections	Resolution recovery, attenuation correction
Additional consideration	
Initial staging/suspected metastases	Full low-dose CT

## Data Availability

No new data were created or analyzed in this study.
